# Divergence of AMP Deaminase in the Ice Worm *Mesenchytraeus solifugus* (Annelida, Clitellata, Enchytraeidae)

**DOI:** 10.4061/2009/715086

**Published:** 2009-07-05

**Authors:** Roberto Marotta, Bradley R. Parry, Daniel H. Shain

**Affiliations:** ^1^Department of Biology, University of Milano, via Celoria 26, 20133 Milano, Italy; ^2^Department of Biology, Rutgers The State University of New Jersey, 315 Penn Street, Science Building, Camden, NJ 08102, USA

## Abstract

Glacier ice worms, *Mesenchytraeus solifugus* and related species, are the largest glacially obligate metazoans. As one component of cold temperature adaptation, ice worms maintain atypically high energy levels in an apparent mechanism to offset cold temperature-induced lethargy and death. To explore this observation at a mechanistic level, we considered the putative contribution of 5′ adenosine monophosphate deaminase (AMPD), a key regulator of energy metabolism in eukaryotes. We cloned cDNAs encoding ice worm AMPD, generating a fragment encoding 543 amino acids that included a short N-terminal region and complete C-terminal catalytic domain. The predicted ice worm AMPD amino acid sequence displayed conservation with homologues from other mesophilic eukaryotes with notable exceptions. In particular, an ice worm-specific K188E substitution proximal to the AMP binding site likely alters the architecture of the active site and negatively affects the enzyme's activity. Paradoxically, this would contribute to elevated intracellular ATP levels, which appears to be a signature of cold adapted taxa.

## 1. Introduction

Glacier ice worms *Mesenchytraeus solifugus * [1] and ssp. *M. solifugus rainierensis * [2] are annelid worms belonging to the family Enchytraeidae (Clitellata: Annelida) [3]. Ice worms are typically found on Pacific coastal glaciers in North America between Oregon and Alaska [4], but a related species has also been reported in Tibet [5]. Ice worms are the largest glacially obligate metazoan that completes their life cycles in glacier ice/snow [6].

Interestingly, ice worms maintain unusually high energy levels (i.e., 5′ adenosine triphosphate, ATP) compared to their mesophilic counterparts, which has been interpreted as a possible mechanism to offset cold temperature-induced lethargy and death [7–9]. Specifically, elevated adenylate levels may counter inherent reductions in molecular motion and enzyme kinetics at low physiological temperatures by increasing the probability of molecular collisions. Indeed, ice worms respond to temperature change by increasing adenylate levels as temperatures fall well below 0°C [7].

To explore this phenomenon, we considered the putative role(s) of two AMP degradative enzymes, AMP phosphatase (AMPP) and AMP deaminase (AMPD), which indirectly control the adenylate pool size by adjusting the rate of AMP degradation [10]. Since relative AMP concentrations change more dramatically than do ADP or ATP, it is logical for any system that monitors cellular energy status to respond to variations in AMP [10, 11]. AMPP represents a major AMP degradative enzyme in eukaryotes but, curiously, we have been unable to detect its presence in ice worms despite extensive efforts. Ice worm AMPD, on the other hand, appears relatively abundant in adult, whole animal specimens. AMPD (EC No 3.5.4.6) catalyses the irreversible hydrolysis of 5′ adenosine monophosphate (AMP) to form inosine monophosphate (IMP) and ammonia [12]. Sequence comparisons across animal phyla demonstrate that AMPD has a highly conserved C-terminal catalytic domain, and a divergent N-terminal region with probable roles in isoform-specific catalytic properties, protein-protein interactions, and subunit associations [13, 14].

To examine the structure and putative role of AMPD in ice worm energy metabolism, we cloned cDNAs by degenerate PCR using an ice worm cDNA library as template. We isolated a combined fragment 543 amino acids in length that included the complete AMPD catalytic region [14], and a 69 amino acid N-terminal region. The predicted ice worm AMPD amino acid sequence was compared with respective homologues across eucaryotic phyla. Although general conservation of ice worm amino acid residues was observed throughout the coding sequence, a few substitutions occurred proximal to highly conserved binding sites; in particular, an ice worm-specific amino acid substitution near the AMP binding site (i.e., K188E) may negatively alter the enzyme's activity and paradoxically contribute to atypically high energy levels observed in glacier ice worms.

## 2. Materials and Methods

### 2.1. Specimens

*Mesenchytraeus solifugus * specimens were collected on Byron Glacier, Alaska, USA, and maintained as described [15]. *Enchytraues albidus * and *Lumbriculus variegatus * were purchased from the Bug Farm (San Rafael, Calif, USA), and maintained as described [16].

### 2.2. cDNA Construction and PCR (Polymerase Chain Reaction)

Total RNA was isolated from ~100 worms of each species as described [17]. cDNA was synthesized with a SMART cDNA construction kit as specified by the manufacturer (Clontech). The following degenerate primer set was used to amplify AMPD: 5′ AARTAYAAYCCNRTNGGNGMN 3′, 5′ CATNGCDATNSSDATYTGNGY 3′ (834 bp expected size). Touchdown PCR (drop of 0.2°C annealing temperature per cycle) was performed with Titanium Taq DNA polymerase (Clontech) using the following conditions: 94°C (10 seconds); 53 → 46°C (1 minute); 72°C (1 minute) for 35 cycles. The 5′ end of ice worm AMPD was amplified by RACE-PCR (rapid amplification of cDNA ends by PCR) using nested, gene-specific primers (5′ GCCTTTATGATTTCTGCAAAG 3′-outside; 5′ CTCTCCCACAGGGTTGTAC 3′-inside), and a Clontech anchor primer (5′ Sequencing primer). Oligonucleotides were synthesized commercially (Sigma-Genosys).

### 2.3. cDNA Library Screening

Independent probes comprising ice worm AMPD cDNAs were constructed with a Prime-a-Gene labeling kit (Promega), incorporating ^32^[P]-*α*dCTP (Perkin-Elmer). Approximately 500 000 phage from a *λ*Triplex2 whole animal ice worm cDNA library were plated and screened at high stringency, as described [18]. Positive *λ* plaques were cored from agar plates with a glass pipette and subjected to Cre-lox-mediated in vivo excision (Clontech). Plasmid DNA was purified using a Wizard Plus SV Minipreps DNA Purification System (Promega). Following restriction endonuclease digestion with *Eco * R1, *Xba * I, and *Hin *d III, inserts ≥600 bp were DNA sequenced (Northwoods DNA, Inc; Becida, Minn, USA). DNA sequence chromatograms were analyzed by Vector NTI 10.3.0 (Invitrogen Corporatioon) and GenBank BLAST analysis [19]. Linear amino acid sequences were aligned with Clustal X software [20]. Newly sequenced AMPD GenBank accession numbers are *Mesenchytraeus solifugus * (EU624492); *Enchytraeus albidus * (EU624493); *Lumbriculus variegatus* (EU624494).

### 2.4. DNA Sequence Verification for Position K188E

AMPD-specific forward and reverse primers (ATGCGGACAGAAACACGTTCCA and TCCGAGTAGACGTTGTTTGCGA, resp.) were employed to amplify a fragment of AMP deaminase from genomic DNA and cDNA encoding the ice worm-specific K188E substitution. Genomic DNA was extracted from ice worm specimens collected from Byron Glacier, Alaska, USA, as described [4], and previously constructed ice worm cDNA libraries [18] were used as templates in cDNA reactions. Standard PCR reactions were conducted with the following parameters: 94° (2 minutes 30 seconds) followed by 30 cycles of 94° (30 seconds); 64° (40 seconds) 72° (1 minute). Reaction products (~650 bp from genomic DNA; ~260 bp from cDNA) were gel-purified and sequenced with the negative strand AMPD specific primer, TGCTGTCTTCAAGGTCGTGCAT (Genewiz, South Plainfield, NJ, USA).

### 2.5. Amino Acid Composition Analysis

Positions that contained an identical amino acid in both ice worm and a mesophilic counterpart were excluded from the comparison. In variable positions (i.e., different amino acids present among mesophilic counterparts, all of which differed from the ice worm sequence), the most abundant amino acid was chosen; in the absence of a dominant residue, the *Drosophila melanogaster * sequence served as default in “animal” comparisons, and *E. albidus * was default in clitellate comparisons. Amino acid compositions, molecular weights, and hydrophobicities were calculated with ProtParam [21].

### 2.6. Protein Structural Analysis

The Protein Database [22] was employed to locate crystallized protein structure templates for the construction of predicted candidate protein models using Swiss Model in the ExPASy server [23, 24]. The crystal structure of *Arabidopsis thaliana * adenosine 5′-monophosphate deaminase (AMPD; chain A) in complex with coformycin 5′ phosphate (an allosteric phosphate ion inhibitor) and the catalytic Zinc ion (in 3.3 Å resolution; PDB: 2A3L) was used as a template structure to generate the 3D model of ice worm AMPD. This was the only AMPD crystal structure available at PDB, but the sequence identity between ice worm and *A. thaliana * AMPD (307 out of 535 amino acids; ~58%) is sufficient for supporting model accuracy. The presence of coformycin 5′ phosphate is unlikely to alter the AMPD 3D structure since coformycin 5′ phosphate, a well-known AMPD and ADA transition state analogue inhibitor [25], is structurally very similar to AMP and is thought to interact in the same way inside the AMPD catalytic site [26]. Ice worm-specific amino acid changes and predicted tertiary structures were manipulated in the Swiss-Pdb Viewer [27] and PyMol [28] to highlight amino acid changes within structures. The stereochemistry quality of ice worm AMPD was validated using PROCHECK [28].”

AMP deaminase mutant models were prepared by introducing K480E and E188K substitutions in the *Arabidopsis thaliana * (PDB ID 2A3L chain A) and *M. solifugus * AMP deaminase linear amino acid sequences, respectively (position 480 in *A. thaliana * is equivalent to 188 in the ice worm). Swiss Model in the ExPASy server [23, 24] was used to model mutant sequences to the template AMP deaminase crystal structure (PDB ID 2A3L chain A). Surface area of the substrate binding plane was defined by four residues within 3.1 Å proximity (K173, Y174, D444, D445) to coformycin 5′-phosphate as determined by PyMol [28], and was calculated in all models by the sum of the surface area of the two triangles composing the substrate binding plane.

## 3. Results

### 3.1. Identification of Ice Worm AMP Deaminase and Homologues

**Figure 1:**
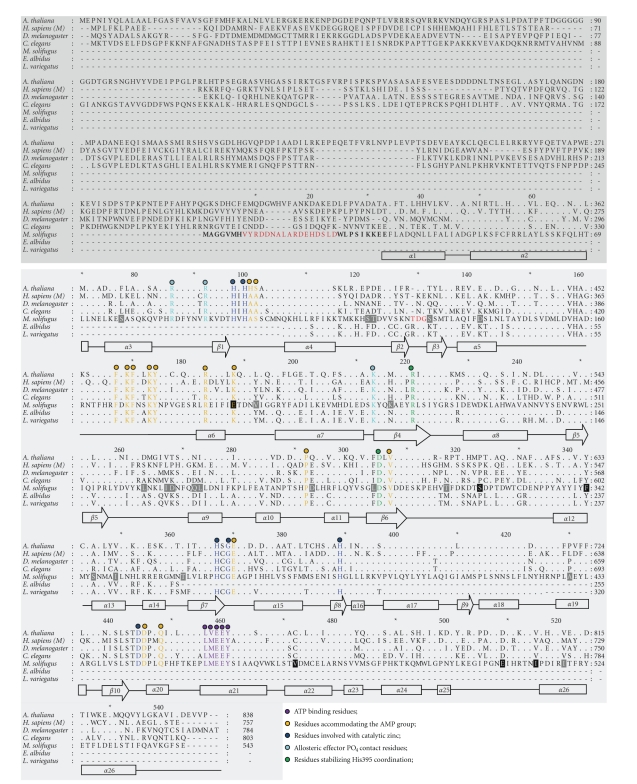
Amino acid alignment of AMP deaminase homologues. The full-length ice worm (*Mesenchytraeus solifugus*) AMPD fragment obtained in this study is represented (543 amino acids). N-terminal domains are shaded dark-gray; C-catalytic domain is shaded light gray. Periods represent conserved residues with respect to *M. solifugus * AMPD. Dashes indicate gaps in the linear amino acid sequence. Gray boxes represent ice worm-specific amino acid substitutions, and black boxes represent unique ice worm substitutions. Secondary structures are represented as *α*-helices (boxes) or *β*-sheets (arrows). Residues denoted by colored spots are explained in the legend at the bottom of the alignment. Ice worm-specific amino acid loops (V8-D24 and T129-G131, see text) are highlighted in red. GenBank accession numbers are *Arabidopsis thaliana * (NP 565886); *Homo sapiens * (NP 000027); *Drosophila melanogaster * (AAF48329); *Caenorhabditis elegans * (NP 001040752); *Mesenchytraeus solifugus * (EU624492); *Enchytraeus albidus * (EU624493), *Lumbriculus variegatus* (EU624494).

A 2,493 bp cDNA representing ice worm AMP deaminase (AMPD) was assembled with overlapping fragments obtained by degenerate PCR, RACE-PCR, and library screening. The combined sequence encoded 543 amino acids of AMPD, including a 69 amino acid N-terminal region, and a 474 amino acid C-terminal catalytic domain containing active sites for AMP binding and regulatory binding sites for the allosteric effectors ATP and inorganic phosphate (Figure 1). Homologous AMPD gene fragments were cloned from two mesophilic annelids (*E. albidus*, *L. variegatus*), and retrieved from GenBank for *Homo sapiens * (Chordata), *D. melanogaster * (Arthropoda), *Caenorhabditis elegans * (Nematoda) and the plant *Arabidopsis thaliana*, from which the only AMPD structural model was available [26]. AMPD homologues displayed >50% sequence identity across all taxa; ice worms were most similar to their clitellate, mesophilic counterparts (~73%) and *D. melanogaster * (~72%), and most distant from *Homo sapiens * (isoform 3) and *A. thaliana * (~58% and ~57%, resp.; Table 1). Insertions at positions 129 (T) and 129 (R) characterized clitellate AMPD homologues (Figure 1). The ice worm AMPD N-terminal domain was considerably more divergent than the C-terminus: identity among animal homologues within the C-terminal domain ranged from 57–73%, while N-terminal domain values were 7–16% (Table 1).

Based on these alignments, we defined ice worm-specific substitutions as those positions at which no other AMPD homologue contained the same amino acid as the ice worm sequence, and at least 50% of the available homologues contained the same amino acid. For example, D122T identifies the dominant mesophilic amino acid at position 122 as D (i.e., six out of seven available homologue sequences encode D at position 122), while ice worm AMPD encodes T. By these criteria, 26 ice worm-specific substitutions (16 nonconservative changes) were identified in the linear ice worm AMPD sequence, all of which occurred in the C-terminal catalytic region (Figure 1). Among invertebrates, only the nematode *C. elegans * displayed a larger number of nonconservative changes than *M. solifugus*, consistent with its deeply rooted ancestry (Table 1).

### 3.2. Structural Analysis of Ice Worm AMPD

**Figure 2:**
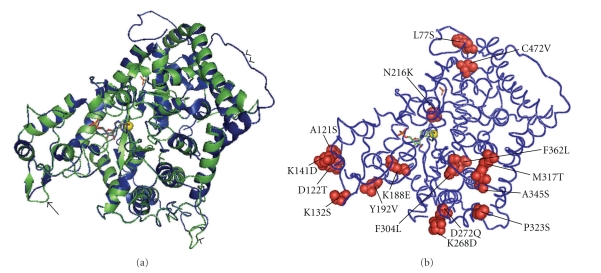
Ribbon diagram of the predicted ice worm AMPD protein. (a) The *M. solifugus * AMPD ribbon diagram (green) is superimposed to the *A. thaliana*-crystal ribbon structure [30] (blue). Double arrowheads point to the large N-terminal transmembrane domain characterizing *A. thaliana * AMPD; single arrowhead points to the ice worm-specific 16 amino acid loop; the arrow points to the ice worm-specific loop connecting strands *β*2 and *β*3. The catalytic zinc is represented by a yellow sphere, the coformycin 5′-phosphate and phosphate ion are depicted as stick models. (b) *A. thaliana*-crystal backbone structure [30] depicting 16 ice worm nonconservative species-specific amino acid substitutions, represented by space filling models.

The linear amino acid sequences of ice worm AMPD homologues were modeled in an effort to predict potential structure/function relationships of ice worm-specific amino acid substitutions. The three-dimensional homology model of the ice worm AMPD enzyme (Figures 2(a) and 2(b)) followed the pattern of the known amidohydrolase [29]. Ice worm AMPD, which contains 26 predicted helices and 10*β* strands, displayed an incomplete triose-phosphate isomerase (TIM) (*α*/*β*)8 barrel fold, with the active site located on the C-terminal side of the imperfect barrel, in a cleft surrounded by multiple helices and loops (Figures 1 and 2(a)). The root mean square deviation between the C*α* atoms from both structures was 0.15 Å, with the largest differences concerning residues not involved in catalysis (Figure 2(a)). Apart from the obvious absence in ice worm AMPD of the large N terminal transmembrane domain, including the “Walker A motif” that characterizes plant AMPD [30], other differences included an ice worm-specific 16 amino acid loop (V8-D24) at the N terminus, and a loop connecting strands *β*2 and *β*3 (T129-G131) (Figures 1 and 2(a)). 

**Figure 3:**
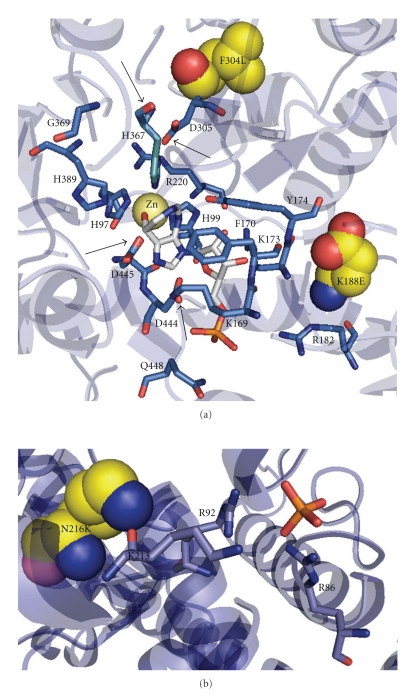
Conformation near the catalytic zinc atom and the AMP-binding pocket, and the phosphate effector site. (a) The zinc atom shows a trigonal bipyramidal conformation ligating coformycin 5′-phosphate, three histidines (H97, H99 and H389), and one aspartic acid (D445). In yellow (space filling model) the specific ice worm substitution F304L. Phe170 and Tyr174 displace the ribose ring of coformycin 5′-phosphate, and Lys169, Lys173, Arg182, Asp444 and Glu448 are able to stabilize the phosphate group of coformycin 5′-phosphate by generating a hydrophilic pocket around this moiety. In yellow (space filling model) the nonconservative ice-worm substitution K188E. Arrows identify residues shifted with respect to those in *A. thaliana * AMPD. The catalytic zinc is represented by a yellow sphere, the coformycin 5′-phosphate and phosphate ion as stick models. (b) Interaction of the phosphate ion with neighboring amino acid residues (Lys213, Arg92 and Arg86). In yellow (space filling model) is the single specific N216K ice worm substitution.

Ice worm AMPD showed general conservation of residues that coordinate the zinc ion, accommodate AMP groups, and bind the allosteric inhibitors ATP and inorganic phosphate (Figure 1). With respect to *A. thaliana * AMPD, slight changes in the position of a few residues involved with the catalytic zinc ion (D305, H367 and D444; Figure 3(a)), and accommodating the AMP (D445; Figure 3(a)) were detected. Additionally, a few nonconservative substitutions occurred within known *α* helices (e.g., L77S, A345S, P430A, C472V), *β* sheets (e.g., N216K) and linker regions (e.g., P323S, K268D) (Table 3). 

Inorganic phosphate is considered as one of the primary physiological inhibitors of AMPD [31, 32], and thus the ice worm-specific N216K substitution proximal to the phosphate allosteric effector site (and also the AMP binding site) may modify electrostatics and structure at these sites (Figure 3(b)).

**Figure 4:**
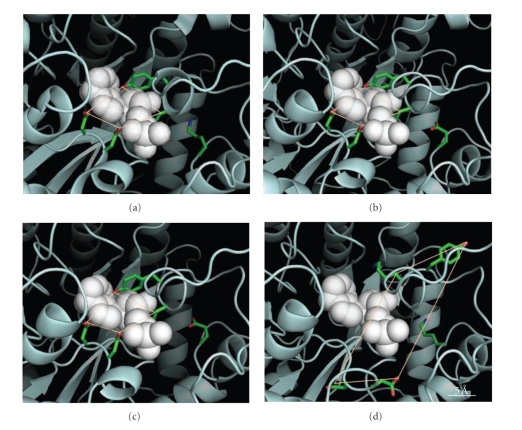
Four sterically constraining residues (K173, Y174, D444 and D445) define the surface area of the AMPD substrate binding plane as ~38 Å^2^ in *A. thaliana * (a), ~36 Å^2^ in *A. thaliana * K188E (b), ~36 Å^2^ in the ice worm E188 (c), and predicted loss of structure in ice worm E188K (d). Position 188 is visible to the right in each panel.

Most dramatic was a K188E ice worm-specific substitution, predicted to occur ~10 Å proximal to the AMP binding site within an already negatively charged pocket. In a comprehensive analysis incorporating >100 known AMPD homologues, all encoded Lys at position 188 while ice worm AMPD contained Glu. The K188E substitution is the result of an A → G nucleotide transition at the first position of codon 188 (Figure 3(a)). We detected this substitution in multiple, independent cDNA clones, and also verified the ice worm change by amplifying the corresponding region of genomic DNA (which contained a 522 base-pair intron following amino acid residue K203). One consequence of the ice worm K188E substitution was a predicted reduction of the substrate binding plane surface area by >5% relative to *A. thaliana * (Figures 4(a)–4(c)); specifically, the K188E substitution was necessary and sufficient for this size reduction in the ice worm's binding pocket. The reciprocal substitution (i.e., E188K) in the context of ice worm sequences, however, did not rescue the substrate binding plane's surface area, but rather distorted the binding plane grossly (Figure 4(d)). Taken together, these point substitutions suggest that position 188 contributes significantly to the architecture of the active site. Note that ice worm-specific substitutions other than K188E likely compensate for the gross distortion observed in Figure 4(d), since the native ice worm structure is clearly similar to that of *A. thaliana* AMPD (cf. Figures 4(a)–4(c)).

### 3.3. Amino Acid Compositional Analysis

To gain a perspective on the types of amino acid substitutions favored in ice worm AMPD, its amino acid sequence was compared to “consensus” sequences (i.e., the dominant amino acid residue at each position) from clitellates and other mesophilic eukaryotes (Table 2). In comparison with representative animal AMPD homologues, ice worm AMPD showed net gains of Asp, His, Leu, and Ser; net reductions in Asn, Cys, Glu, Lys, and Met; slight increases in the number of charged and acidic residues; a gain of polar residues in the clitellate comparison; and a reduction in amino acid side chain volumes (i.e., Mwt) and hydrophobic amino acids, more remarkable in the clitellate comparison (Table 2). The most frequent ice worm AMPD residue preferences were V → I, A → S, L → F, and K → D, though no significant directional bias was detected (Table 3). The majority of substitutions occurred in *α*-helices and in loops connecting different secondary structures, while relatively few substitutions occurred in *β*-strands (Table 3). Finally, ice worm AMPD contained fewer H-bonding residues than *A. thaliana* AMPD (185 and 167 amino acids devoid of H bonds, resp.).

## 4. Discussion

Cold adapted enzymes typically display gains in flexibility at the expense of thermal stability [33–35]. Among the 26 ice worm-specific amino acid substitutions deduced in this study, many are likely to increase flexibility based on reduction of the side chain volume (e.g., Y192V, F304L), increasing local hydrophilicity (e.g., L77S, A121S) or loss of well-conserved proline residues (e.g., P323S, P430A), in comparison with mesophilic AMPD counterparts. Flexibility introduced by the single ice worm-specific change F304L (adjacent to D305, which is critical in stabilizing Zn coordination; [30]) may facilitate reactivity of the catalytic zinc critical for deamination through a short-lived tetrahedral intermediate transition state [12, 36]. 

Apart from a slight increase in charged amino acids in ice worm AMPD with respect to its animal counterparts, increased polarity, reductions in side chain volumes and charged residues (e.g., Glu and Lys), and decreased core hydrophobicity (Table 2) are all trends present in cold adapted proteins, and consistent with gains in molecular flexibility [34, 37, 38]. The modest gain in acidic amino acids (i.e., Lys and Arg) has also been observed in other cold adapted enzymes, and explained by Feller et al. [39] as improved solvent interactions that can destabilize the external shell of proteins. The increase in Asp, however, is less obvious; it has the shortest side chain of the charged amino acid and thus destabilizes a protein by forming ionic bonds less easily [37]. Additionally, the observed reduction in H-bond number in ice worm AMPD is consistent with strategies to increase backbone flexibility [35]. Likewise, gains in flexibility have been reported to occur particularly at *α*-helical segments [40], and several ice worm-specific AMPD substitutions occurred within loops and helices (Table 3).

### 4.1. Ice Worm-Specific Substitutions That May Affect Enzyme Activity

Position 188 in AMPD is located at the center of a negatively charged pocket, and the addition of yet another negative charge (i.e., K188E in ice worms) could introduce additional destabilizing forces owing to negative-negative repulsion. Since the substitution lies adjacent to the active site, the effect of repelling charges may indeed introduce flexibility to the enzyme's active site, contributing to its catalytic efficiency at low temperatures. However, another explanation for the nonconservative K188E substitution should be considered. Bae and Phillips [41] and Fields [42] have hypothesized and empirically validated that substitutions within the active site of enzymes performing homologous functions, varying only in temperature optima, will not occur. While residue 188 has been proposed to accommodate the AMP ribose and phosphate groups [43], our model based on *A. thaliana * AMPD suggests that ice worm residue 188 does not function in such a faculty. Rather, K188E is 10–15 Åfrom the AMP binding pocket and interacts with a loop of decreased flexibility (owing to P176) responsible for coordinating AMP. In fact, comparison of the *A. thaliana * structure (Figure 4(a)) with the *A. thaliana * K188E AMPD model (Figure 4(b)) supports a loss of area in the active site due to K188E (comparable with the calculated area of the ice worm's active site; Figure 4(c)). Due to these topological considerations, K188E may induce an architectural change of the active site in ice worm AMPD, raising interesting evolutionary questions (mentioned in what follows).

### 4.2. Evolutionary Considerations

The quest to understand the molecular adaptations of proteins to cold environments usually centers on the assumption that the psychrophilic protein should function more efficiently than its mesophilic counterpart at lower temperatures. For ice worm AMPD, however, this may not necessarily be the case. The reaction catalyzed by the AMPD represents a branch-point in the energy-generating adenylate catabolic pathway regulating the availability of adenosine nucleotides: blocking this pathway reduces the depletion of the total adenine pool and thus the metabolically expensive requirement for the de novo synthesis of ATP [12]. Ice worms (and other psychrophiles) paradoxically increase ATP levels with falling temperatures [7, 8], and the mechanism underlying this response appears to be dependent on the cell's ability to degrade AMP [9, 10]. Consistent with this idea, knocking out the major AMP degradative enzyme in bacteria (5′ AMP nucleosidase) resulted in an ~30% increase in steady-state ATP levels in the mutant strain, which was significantly more cold tolerant than wild type [44].

Thus the putative mechanism by which ice worms increase their cellular ATP levels involves a predicted reduction in the ability to remove AMP from the adenylate pool, and subsequent activation of ATP synthetic processes as a direct consequence of increased AMP levels [10, 11]. Our ongoing studies suggest that the major eucaryotic AMP degradative enzyme, AMP phosphatase, may be silenced in ice worms and, based on the current study, that the alternate AMP degradative pathway (i.e., AMP → IMP via AMPD) may not function efficiently. Specifically, nonconforming ice worm-specific substitutions in AMPD—primarily K188E—would arguably decrease the enzyme's catalytic activity by altering the architecture of the active site. However, ice worms have maintained expression of the AMPD gene over evolutionary time, as evidenced by its abundance in our cDNA libraries, and so AMPD must have some function in ice worm energy metabolism. Possibly, ice worms have shifted their energetic “thermostat” by dramatically reducing their ability to degrade AMP, but still retain the potential to remove AMP from the adenylate pool should ATP levels become excessively high.

## Figures and Tables

**Table 1:** Comparison of sequence similarity, GC content and species-specific amino acid substitutions among AMPD homologues. Nucleotide and amino acid (aa) percentages were calculated by BLAST [19] comparisons of each homologue sequence to its respective ice worm counterpart.

Homologues	*M. solifugus*	*M. solifugus*	*M. solifugus*	*M. solifugus*	GC (%)	Specific/nonconservative amino acid substitutions
**AMP-deaminase**	Similarity (%) sequence nucleotide	Similarity (%) sequence aa identical/aa similar	Similarity (%) to C-domain aa identical/aa similar	Similarity (%) to N-domain aa identical/aa similar		
*A. thaliana*	57	58/75	60/76	7/12	42.6	94/51
*H. sapiens*	59	57/77	57/76	13/19	45.0	89148
*D. melanogaster*	67	72/86	73/87	14/21	53.5	25/11
*C. elegans*	62	66/81	66/82	16/19	43.9	41/16
*M. solifugus*	—	—	—	—	48.3	26/16
*E. albidus*	65	71/85	—	—	455	0/0
*L. variegatus*	69	74/86	—	—	46.8	3/0

**Table 2:** Amino acid composition changes in ice worm AMPD. Number indicate gains (+) or losses (−) in ice worm AMPD in comparison with sequences among clitellates (*E. albidus, L. variegatus*) and other mesophilic eucaryotes (*A. thaliana*, *H. sapiens*, *D. melanogaster*, *C. elegans*). Molecular weight change (Mwt) is in Dalton (Da).

Residues	Plant	Mesophilic eucaryotes			Mesophilic clitellates			
	*A. thaliana*	*H. sapiens*	*D. melanogaster*	*C. elegans*	Animal	*E. albidus*	*L. variegatus*	Clietallates
Ala	0	−5	−2	+3	−1	−3	−2	−3
Arg	−1	+4	0	−1	+1	0	−1	−1
Asn	+3	+1	−9	−5	−4	+1	+2	+2
Asp	+1	+6	+8	+1	+5	+4	+4	+4
Cys	−2	−4	−4	−1	−3	−2	−2	−2
Gln	−3	−1	−5	+1	−2	0	0	0
Glu	+2	−5	−3	−4	−4	−2	−2	−2
Gly	−2	−1	+2	−2	0	−2	−3	−3
His	−2	+3	+7	+1	+4	+1	+1	+1
Ile	−3	+1	−3	+4	+1	+2	+2	+3
Leu	0	+7	+6	0	+4	+5	+7	+6
Lys	−2	−8	+1	−4	−4	−2	−1	−2
Met	+6	−6	−6	+3	−3	−1	−1	−1
Phe	0	−4	+1	+1	−1	−3	−4	−4
Pro	0	−1	−3	+1	−1	−1	−1	−1
Ser	+7	+8	+9	+7	+8	+3	+3	+3
Thr	+2	+1	+1	0	+1	+3	+2	+3
Trp	−1	+1	0	0	0	0	0	0
Tyr	0	0	−5	0	−2	−2	−2	−2
Val	−7	+5	0	−4	0	−3	−2	−3
Polar	+5	+5	0	−5	0	+4	+5	+5
Charged	−2	0	+13	−7	+2	+1	+1	+1
Acidic	+3	+1	+5	−3	+1	+2	+2	+1
pl	+7.18	+6.88	+6.6	+6.51	—	+6.16	+6.56	—
Δpl	−0.83	−0.53	−0.25	−0.16	−0.31	−0,48	−0,46	−0.47
Mwt.	−296,6	−128.6	−1010	75.9	−319	−273,5	−96,2	−185
Hydrophobicity	−7	−3	−5	+6	−1	−8	−5	−5
Aliphatic Index	−5,91	+7.7	+2.72	+1.2	+4	+6.7	+8^.^5	+8

**Table 3:** Abundant ice worm-specific amino acid substitutions. pa is the random probability of a directional bias equal to or greater than that observed (calculated using the two-tail binomial distribution). L, H, and S are, respectively, loop, *α*-helix and *β*-sheet; i, internal; e, external.

Substitutions	Forward	Reverse	Position	Region	pa
V → I	4	0	267,349,515,520	Le-Hi-Le-Hi	0.125
A → S	2	0	121,345	Le-Hi	0.5
L → F	0	2	304,362	Si-Li	0.5
K → D	2	0	141,268	He-Le	0.5
L → S	1	0	77	He	—
K → S	1	0	132	Se	—
P → S	1	0	323	Le	—
I → L	1	0	273	Hi	—
F → L	1	0	304	Si	—
D → T	1	0	122	Le	—
M → T	1	0	317	Li	—
P → A	1	0	430	Hi	—
C → V	1	0	472	Hi	—
N → K	1	0	216	Si	—
K → E	1	0	188	Li	—
Y → F	1	0	342	Li	—
D → Q	1	0	272	Hi	—
A → L	1	0	263	Le	—
